# Supplementary material for: Analyzing inter-reader variability affecting deep ensemble learning for COVID-19 detection in chest radiographs
Source: PLoS One. 2020 Nov 12;15(11):e0242301. doi: 10.1371/journal.pone.0242301 (PMC7660555; doi:10.1371/journal.pone.0242301)
Supplement: S1 File — (DOCX) [file pone.0242301.s001.docx]

SUPPLEMENTARY MATERIAL

# INTER-READER VARIABILITY ANALYSIS USING STAPLE

The Simultaneous Truth and Performance Level Estimation (STAPLE) algorithm is summarized as follows: Let *Q = (q_1_, q_2_, …., q_n_)^N^* and *R = (r_1_, r_2_, …., r_n_)^N^* denote two column vectors, each containing *A* elements. The elements in *Q* and *R* represent sensitivity and specificity parameters, respectively, characterizing one of *N* segmentations. Let *D* denote an *M × N* matrix that describes segmentation decisions made for each image pixel. Let *N* denote an indicator vector containing *M* elements representing hidden, true binary segmentation values. The complete data can be written as (*D, N*) and the probability mass function as *f (D, N|q, r)*. The performance level of the experts, characterized by a tuple (*q, r*) is estimated by the EM algorithm, which maximizes (*q’, r’*), the data log-likelihood function, given by,

|  | $\left( q^{'}, r^{'} \right)={argmax}_{q, r}ln(f\left( D,N \vert q, r) \right)$ | (1) |
| --- | --- | --- |
|  |  |  |

We used the following performance measures including Kappa statistic, sensitivity, specificity, positive predictive value (PPV), and negative predictive value (NPV) to analyze inter-reader variability and assess program performance. We used the STAPLE-generated consensus ROI as to the standard reference and measured its agreement with that generated by the top-N ensembles and the annotations of Rad-1 and Rad-2. We propose an algorithm to determine the set of True Positive (TP), False Positive (FP), True Negative (TN), and False Negative (FN) for different IoU thresholds in the range (0.1 – 0.7). The IoU evaluation metric, also called the Jaccard Index, is widely used in object detection, given by a ratio as shown below:

|  | $IoU (Jaccard Index)= \frac{Area of overlap}{Area of union}$ | (2) |
| --- | --- | --- |

where *Area of overlap* measures the overlap between ROI annotations and *Area of union* denotes their total combined area. An annotated ROI provided by a given radiologist or that predicted by the top-N ensemble is considered as a TP if the IoU with the STAPLE-generated consensus ROI is greater than or equal to a given IoU threshold. Each radiologist or top-N ensemble predicted ROI that produces an IoU less than the threshold or falls outside the consensus ROIs is counted as FP. The FN is defined as a radiologist ROI or that predicted by the top-N ensemble that is completely missing when there is an ROI in the consensus ROI. If there is an image with no ROIs on both the masks under test, then we consider it as TN. The values are determined at ROI-level per image and summed to calculate the Kappa statistic given by,

|  | $Kappa=1- \frac{{1-p}_{o}}{{1-p}_{e}}$ | (3) |
| --- | --- | --- |
|  |  |  |

Here, *p_o_* is the measure of relative observed agreement and *p_e_* denotes the agreement through the hypothetical probability of chance. The values of *p_o_* and *p_e_* are computed as follows:

|  | | | $p_{o}= \frac{(TP+TN)}{TP+FN+FP+TN}$ | (4) |
| --- | --- | --- | --- | --- |
|  | | | $p_{e}= \frac{p\_true}{p\_false}$ | (5) |
|  | | $p\_true= \frac{\left( TP+FN \right)(FP+TP)}{\left( TP+FN+FP+TN \right)^{2}}$ | | (6) |
|  | $p\_false= \frac{\left( FP+TN \right)(FN+TN)}{\left( TP+FN+FP+TN \right)^{2}}$ | | | (7) |
|  |  | | |  |

The sensitivity, specificity, PPV, and NPV parameters are defined as,

|  | $Sensitivity= \frac{TP}{FN+TP}$ | (8) |
| --- | --- | --- |
|  | $Specificity= \frac{TN}{FP+TN}$ | (9) |
|  | $PPV= \frac{TP}{FP+TP}$ | (10) |
|  | $NPV= \frac{TN}{FN+TN}$ | (11) |
|  |  |  |

Kappa values of 1 and 0 denote complete agreement and disagreement (other than occurring by chance) among the readers, respectively. The value of Kappa becomes negative if the agreement gets worse than random. The algorithm for measuring inter-reader variability is given below, where *m^1^*, *m^2^*, and *m^p^* denote the ROI annotations of Rad-1, Rad-2, and that predicted by the top-N ensemble, respectively.

**Algorithm to assess inter-reader variability and program performance.**

| **Algorithm** |
| --- |
| 1: **Input:**  Data $\left\{ m^{1},m^{2},m^{p} \right\}$, Threshold $thr$  2: **for**$i=0,1,2,\ldots, N$ **do**  3: $m_{i}^{ref}=staple(m_{i}^{1},m_{i}^{2})$  4: **if** ($m_{i}^{ref}$ **or** $m_{i}^{1}$/$m_{i}^{2}/m_{i}^{p}$) contains ROIs **then**  5: **for** $ROI_{j}$in $m_{i}^{1}$/$m_{i}^{2}/m_{i}^{p}$ **do**  6: **for** $ROI_{k}$ in $m_{i}^{ref}$ **do**  7: $metric=IOU(ROI_{j},ROI_{k})$  8: **if** $metric\geq thr$ **then**  9: $TP\leftarrow TP+1$  10: **else** **if** $0<metric<thr$ **or** missing $ROI_{k}$ **then**  12: $FP\leftarrow FP+1$  13: **else** **if** missing $ROI_{j}$ **then**  14: $FN\leftarrow FN+1$  15: **else**  16: $TN\leftarrow TN+1$  17: **end for**  18: **Measure and** **output:** $Kappa, Sensitivity, Specificity, PPV, and NPV$ |

# CRM VISUALIZATION

In this study, we use the CRM [15] visualization method to interpret the learned behavior of individual models and their ensembles in localizing COVID-19 viral disease-specific ROI manifestations. The method has been shown to deliver better localization performance than class-activation mapping (CAM)-based visualization. CRM-based localization considers the fact that a feature map spatial element from the trained model’s deepest convolution layer would not only contribute to increasing the prediction score for an expected class but also decreasing the score for other class outputs. This helps in maximizing the gap between the scores for various classes. The process results in highly-discriminative ROI localization since it uses the incremental mean-squared error (MSE) measured from the output nodes. We construct an ensemble of CRMs by averaging those generated from various fine-tuned models for COVID-19 detection. The size of CRMs from individual models is up-scaled to the size of the image input through a normalization process. This is because the CRMs vary in size depending on the feature map dimensions from the deepest convolutional layer of the individual models. Based on empirical observations, the CRMs are thresholded to remove mapping scores below 20% to alleviate noise resulting from low mapping scores when constructing CRM ensembles. The resulting ensemble CRM localization is expected to compensate for the error of missed ROI by individual models and enhance COVID-19 disease ROI localization.

# EMPIRICALLY DETERMINED FEATURE EXTRACTION LAYERS

The Candidate CNN layers delivering superior classification performance during the first stage of CXR-specific pretraining is shown below. The naming conventions for the layers are based on the Keras DL framework.

| **Model** | **Truncated layers** |
| --- | --- |
| VGG-16 | Block5-conv3 |
| VGG-19 | Block5-conv4 |
| Inception-V3 | Mixed3 |
| Xception | Add-3 |
| DenseNet-121 | Pool3-pool |
| MobileNet-V2 | Block-9-add |
| NASNet-mobile | Activation-94 |
| ResNet-18 | Add-6 |

# T-SNE VISUALIZATION OF FEATURE EMBEDDING

We used t-SNE to visualize the embedding of the 1024-dimensional feature space into 2 dimensions, as shown below. It is observed that the feature space for the normal and COVID-19+ classes is well-separated and clustered to facilitate the classification task.


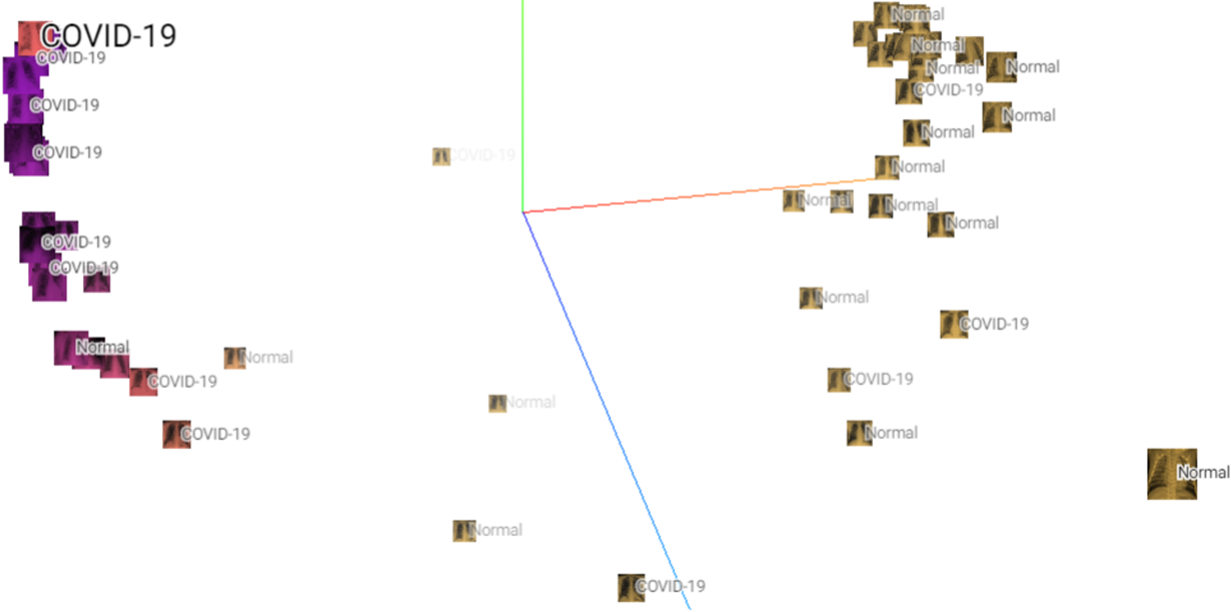


**Visualizing feature embedding for the ResNet-18 fine-tuned model using t-SNE.** The plot shows a 1024-dimensional space embedded into 2 dimensions.

# PR CURVES FOR THE TOP-PERFORMING INDIVIDUAL MODELS

The precision-recall (PR) curves of the best performing models using Rad-1, Rad-2, and the STAPLE-generated consensus ROI are shown below.


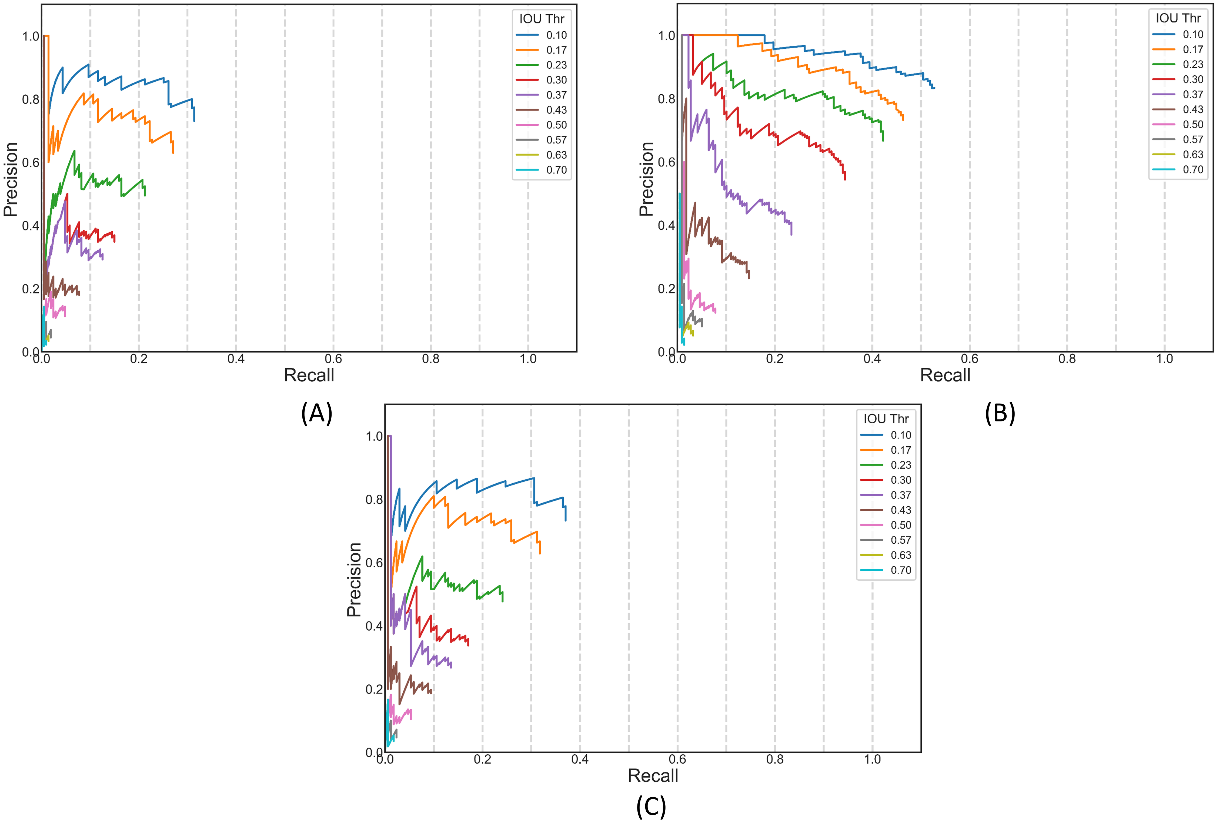


**PR curves for the top-performing models using ROI annotations.** (A) Rad-1; (B) Rad-2; (C) STAPLE-generated ROI consensus.

# PR CURVES FOR TOP-N ENSEMBLE CRMs

The PR curves obtained with the top-N ensemble CRMs using Rad-1, Rad-2, and STAPLE-generated consensus ROI are shown below.


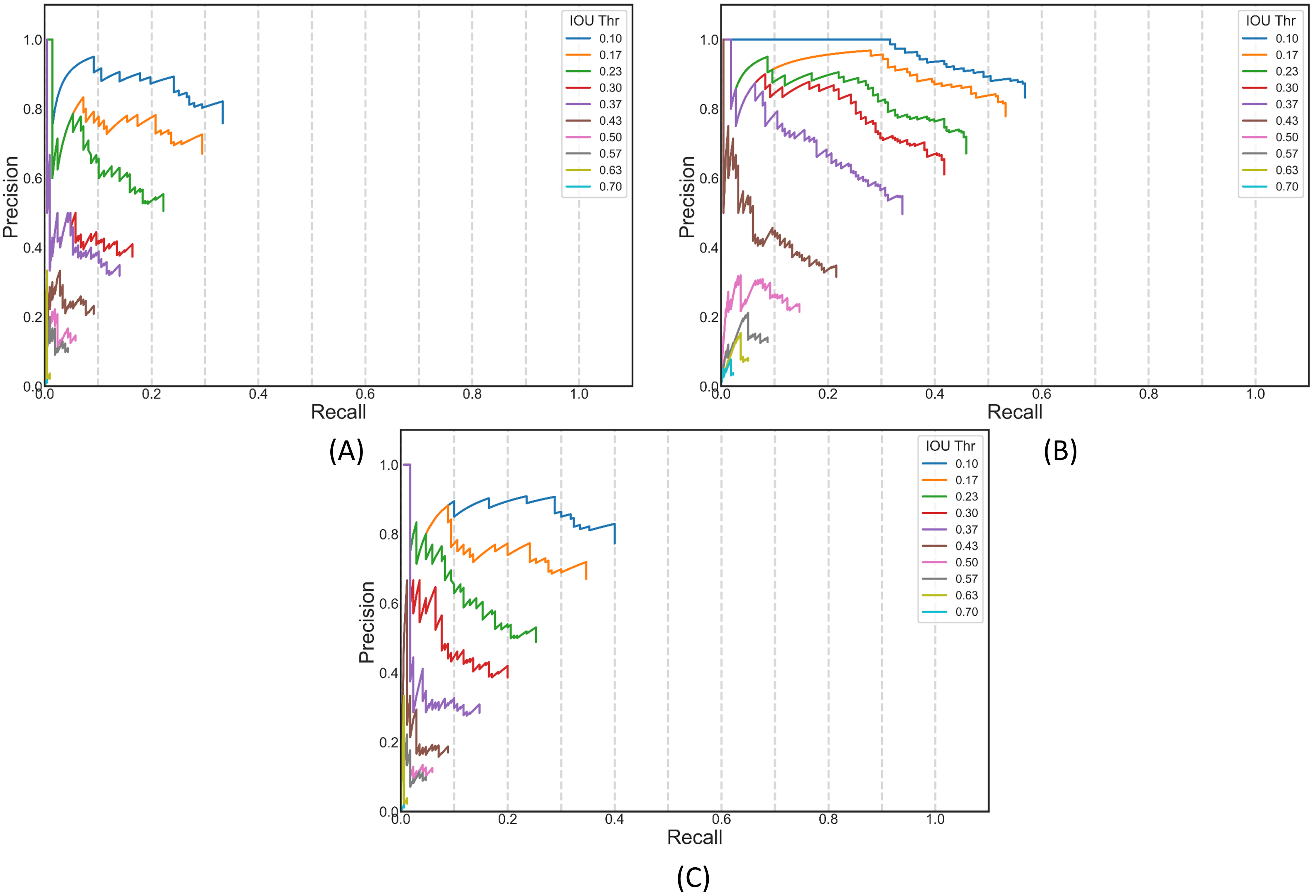


**PR curves for the top-N performing models using ROI annotations.** (A) Rad-1 (N = 3); (B) Rad-2 (N = 5); (C) STAPLE-generated consensus ROI (N = 3).
