# Supplementary material for: Analyzing inter-reader variability affecting deep ensemble learning for COVID-19 detection in chest radiographs
Source: PLoS One. 2020 Nov 12;15(11):e0242301. doi: 10.1371/journal.pone.0242301 (PMC7660555; doi:10.1371/journal.pone.0242301)
Supplement: Attachment — Submitted filename: PONE-D-20-22486R1_Response to Reviewers.docx [file pone.0242301.s003.docx]

**Manuscript ID: PONE-D-20-22486R1**

**Article Title: “Analyzing inter-reader variability affecting deep ensemble learning for COVID-19 detection in chest radiographs”**

**To:** Dr. Yuankai Huo

**Re:** Response to reviewers

Dear Editor,

Thank you for arranging peer-review of our submission, and to the reviewers for their time and effort for providing us their critical comments. My co-authors and I are grateful for encouraging resubmission of our manuscript and the opportunity to address reviewer comments. We would be delighted for our research article to appear in the highly respected PLOS ONE journal. As suggested, we have made the following changes to the revised manuscript:

1) **The demographic information provided by the data providers for the various datasets used in this study are included.**
2) **The data distribution tables are merged.**
3) **Certain non-essential content have been moved to the supplementary material to enable the readers to concentrate on the main ideas.**
4) **The figures were checked with the PACE digital diagnostic tool to ensure they are in compliance with PLOS requirements.**

In the resubmission, we include (a) our point-by-point response to the comments (below in “Response to Reviewers”); (b) a marked-up copy of the manuscript that highlights changes made to the original version; and, (c) a clean updated manuscript without highlights (PDF main document).

On a personal note, we wish you, the members of the PLOS ONE office staff, and the peer-reviewers good health in these difficult times caused by the COVID-19 pandemic.

Sincerely,

Sivaramakrishnan Rajaraman, PhD

National Library of Medicine

National Institutes of Health

Bethesda, MD, USA

[sivaramakrishnan.rajaraman@nih.gov](mailto:sivaramakrishnan.rajaraman@nih.gov)

**Response to Reviewers Reviewer#1: Generally, my comments have been addressed. Please provide a demographic table of the patients if possible.**

**Author response:** We render our sincere thanks to the reviewer for the valuable comments and appreciation of our study. As recommended, we have included the demographic information provided by the data providers for the various datasets used in this study as shown below:

**Table 1. Demographic study.** The table shows the statistics such as patient count, age, and sex for the various datasets used in this study. NA denotes Not Available.

| **Dataset** | **Total** | | **Mean (age)** | | **Standard deviation (age)** | |
| --- | --- | --- | --- | --- | --- | --- |
|  | **Male** | **Female** | **Male** | **Female** | **Male** | **Female** |
| NIH [8] | 63340 | 48780 | 47.04 | 46.6 | 17.19 | 16.27 |
| Pediatric CXR [29] | NA | NA | NA | NA | NA | NA |
| RSNA [30] | 17006 | 12888 | NA | NA | NA | NA |
| CheXpert [31] | 132871 | 91007 | 60.83 | 60.43 | 18.19 | 18.19 |
| Montreal-COVID-19 [32] | 131 | 64 | 59.15 | 54.97 | 16.27 | 15.11 |
| Twitter-COVID-19 | 17 | 11 | 13.43 | 19.61 | 8.75 | 8.38 |

**Reviewer#2: The authors satisfactorily addressed my concerns and revised the manuscript correspondingly. The revised paper is still a very long paper, I would recommend to move some non-essential content to supplementary or simplify the content so reader can concentrate on the main ideas (such as the data distribution tables could be merged to one if possible).**

**Author response:** We thank the reviewer for the appreciation and insightful comments on this study. As recommended, we have made the following changes to the revised manuscript:

1. The details pertaining to the datasets and their distribution, used in various stages of learning, are merged into a single table (Table 2) as shown below:

**Table 2. Datasets and their distribution used in various stages of learning**. In the first stage of repeated CXR-specific pretraining, a custom CNN and a selection of ImageNet-pretrained CNNs are retrained on a large selection of CXRs to learn CXR-specific features to categorize them as showing normal or abnormal lungs. During the second stage of repeated CXR-specific pretraining, the first-stage pretrained models are retrained on a collection of CXRs to categorize them as showing normal lungs, bacterial pneumonia, or non-COVID-19 viral pneumonia manifestations. Note that the pediatric CXR dataset predates the onset of the SARS-CoV2 virus, and therefore the viral pneumonia is of non-COVID-19 type. During the COVID-19 detection stage, the second-stage pretrained models are fine-tuned to classify CXRs into showing normal lungs or COVID-19 viral patterns.

| **Dataset** | **Normal** | **Abnormal** | **Bacterial pneumonia** | **Non-COVID-19 viral pneumonia** | **COVID-19+** |
| --- | --- | --- | --- | --- | --- |
| **First stage of repeated CXR-specific pretraining** | | | | | |
| RSNA | 8331 | 17833 | - | - | - |
| CheXpert | 16480 | 17000 | - | - | - |
| NIH | 59892 | 51708 | - | - | - |
| Total | 84703 | 86541 | - | - | - |
| **Second stage of repeated CXR-specific pretraining** | | | | | |
| RSNA | 400 | - | - | - | - |
| CheXpert | 400 | - | - | - | - |
| NIH | 400 | - | - | - | - |
| Pediatric CXR | 1583 | - | 2780 | 1493 | - |
| Total | 2783 | - | 2780 | 1493 | - |
| **COVID-19 detection** | | | | | |
| RSNA | 120 | - | - | - | - |
| CheXpert | 120 | - | - | - | - |
| NIH | 120 | - | - | - | - |
| Montreal-COVID-19 | - | - | - | - | 226 |
| Twitter-COVID-19 | - | - | - | - | 134 |
| Total | 360 | - | - | - | 360 |

2. Per reviewer suggestions, the following information have been moved to the supplementary material (S1 File.pdf) to help the readers concentrate on the main ideas:

1. Inter-reader variability analysis using STAPLE algorithm (Section A of the supplement S1 File)
2. Class-selective relevance (CRM) visualization (Section B of the supplement S1 File)
3. Table showing empirically determined feature extraction layers and its related discussion (Section C of the supplement S1 File)
4. t-SNE visualization of feature embedding (Section D of the supplement S1 File)
5. P-R curves for the top-performing individual models (Section E of the supplement S1 File)
6. P-R curves for top-N ensemble CRMs (Section F of the supplement S1 File)
